# Quantifying Randomness in Stochastic Bit Sequences

**DOI:** 10.3390/s26154825

**Published:** 2026-07-30

**Authors:** Christoph Lange, Andreas Ahrens, Yadu Krishnan Krishnakumar, Denise Engert, Ming Yin

**Affiliations:** 1 School of Engineering—Energy and Information, Hochschule für Technik und Wirtschaft Berlin (University of Applied Sciences), Wilhelminenhofstraße 75 A, 12459 Berlin, Germany; 2 Faculty of Engineering, Hochschule Wismar (University of Applied Sciences: Technology, Business and Design), Philipp-Müller-Straße 14, 23966 Wismar, Germany; andreas.ahrens@hs-wismar.de (A.A.); yadu.krishnakumar@alumnos.upm.es (Y.K.K.); 3 Escuela Técnica Superior de Ingeniería y Sistemas de Telecomunicación (ETSIST), Universidad Politécnica de Madrid, Campus Sur, Calle Nikola Tesla s/n, 28031 Madrid, Spain; 4 Telekom Innovation Laboratories, Deutsche Telekom, Winterfeldtstraße 21, 10781 Berlin, Germany; denise.engert@telekom.de (D.E.); ming.yin@telekom.de (M.Y.)

**Keywords:** randomised bit sequences, burst, test, probability, gap process, gap distribution, burstiness factor

## Abstract

Recent advancements in the field of communications and cryptology have attracted significant research efforts in studying randomness of bit sequences. Randomised bit sequences play a vital role in sensor applications by ensuring security (i.e., protecting against brute-force, replay, and eavesdropping attacks in wireless networks) and reliable signal processing (i.e., in sensor multiplexing schemes such as code-division or time-division schemes). Such bit sequences enable spread-spectrum techniques, which allow an improved signal separation in dense networks such as structural health monitoring. Furthermore, unpredictability is essential for secure communication among sensors, as seen in fiber Bragg grating systems. The mentioned studies have led to the development of different test methodologies, such as the NIST (National Institute of Standards and Technology) test suite, whose main objectives are to verify the independence of the individual elements in the sequence and to test their distribution within the bitstream. In this article, industry-relevant use cases are discussed for the application of random bit sequences and a gap-based approach for analysing bit sequences is presented and used together with a NIST-specified test. We introduce a simplified non-IID test approach (independent and identical distribution) to indicate whether the commonly considered IID characteristics of random variables are violated. To validate the proposed approach, this study employs different polynomial and nonpolynomial sequence generation methods. Furthermore, random sequences generated by different methods in hardware are included in the verification tests. The results confirm that the proposed methods of randomness assessment effectively indicates the non-IID characteristics of randomised bit sequences.

## 1. Introduction

Random sequences are fundamental building blocks in many technical and scientific disciplines as they are able to generate unpredictable patterns, e.g., bit sequences in digital signal processing and transmission systems. They serve as essential components for various technical systems, including estimation tasks in communication systems and cryptographic applications.

In cryptography, random sequences form the basis for encryption algorithms and nonces (numbers used once), as these cannot be reproduced by an attacker. Without randomised bit sequences, cryptographic signatures and public-key systems would lose their security. In communications engineering, random signals constitute the foundation for test signals employed in channel modelling, equalisation, and simulation tasks.

True random signals can be derived from physical sources such as thermal noise, whereas pseudorandom signals are—strictly speaking—deterministically generated numbers, since an underlying algorithm forms the basis for the construction of an output sequence. Nevertheless, from the perspective of an external observer, these numbers statistically resemble true random numbers, especially when the generation mechanism is unknown. However, they are always reproducible using a known algorithm with a known initial seed. Such sequences can be generated efficiently, for example, using linear or nonlinear shift registers [1], but they become predictable if seed and algorithm are known. Moreover, the resulting sequences are often periodic, as elements repeat once a previously used internal state is reached [2,3,4].

Here, randomised sequences that arise from quantum phenomena have attracted a lot of attention in the research field (e.g., sequences generated using equipment from the company ID Quantique, as they are considered as non-deterministic sequences in contrast to shift-register-based random sequences. QRNG (Quantum Random Number Generator) chips are often used in this context, which evaluate, for example, the fluctuation of the photon number from a weak light source [5].

A number of test methods have been established for evaluating the randomness of randomised sequences. These methods attempt to detect, for instance, the distribution of elements within the bitstream or recurring patterns. However, the “unpredictability of random numbers” is still difficult to assess using such tests.

A key requirement is that random sequences at least partially satisfy the IID (independent and identically distributed) assumption, according to which the individual elements of the sequences are independent of each other and exhibit identical probability distributions [2,4].

A comparatively recent approach to the analysis of randomness in binary sequences is based on the statistical characterisation of gaps, defined as the number of zero-valued elements between successive non-zero elements [6]. Parameters derived from this methodology enable a more detailed examination of the internal structure of the sequence under investigation. In particular, the distribution of gap lengths provides an initial indication whether the assumption of independent and identically distributed (IID) samples is satisfied or not. Under ideal IID conditions, gaps of length zero—corresponding to consecutive non-zero elements—are expected to occur with a probability of 0.5 within the whole sequence.

In contrast to conventional tests such as the monobit test [7], which solely evaluates the proportion of zero and non-zero elements, gap-based metrics capture temporal dependencies within the sequence. Specifically, the probability that a non-zero element is immediately followed by another non-zero element can only be inferred through the analysis of the gaps between non-zero elements. This constitutes a key advantage of the gap-based approach, as it provides direct insight into temporal correlations and potential clustering effects within the bitstream.

However, the gap analysis does have limitations. An important precondition is the independence of the gaps and the gap lengths. This is a simplification which is advantageous from the theoretical viewpoint, although under practical conditions, this cannot always be maintained. Despite this limitation, the gap-based approach offers a valuable complementary tool for randomness assessment, particularly for detecting bursty behaviour that may remain hidden from conventional statistical tests.

While numerous test methods concentrate on verifying the IID assumption (e.g., [5,7,8]), this work focuses on testing for potential non-IID-compliant behavior. Such behavior manifests itself when non-zero elements occur in concentrated clusters, i.e., in a bursty manner, so the burstiness factor proposed by Goh and Barabási [9] is employed as an indicator. Accordingly, we examine and test whether the considered random sequences may not be truly random, in other words, we aim to detect non-randomness based on analysing the gaps between neighbouring non-zero elements.

The test objects comprise, on the one hand, real random sequences generated by various hardware-based methods that will be described in detail subsequently, and on the other hand, (pseudo)random sequences algorithmically generated using linear and nonlinear shift registers. The genuine random sequences are those produced in a laboratory setting; they are analysed to investigate their suitability for practical deployment in telecommunications networks, for example, in key distribution for secure telecommunication networks. The pseudorandom sequences are included to ascertain the similarities and differences between genuine and pseudorandom sequences and to establish a link to prior work published in [5,6,10,11].

The novelty of this work lies in using the burstiness of non-zero elements within randomised sequences as a rejection criterion for randomness under the IID framework. For calculating the level of burstiness, the proposed test relies on the gaps between neighbouring non-zero elements. The developed test method demonstrates that measuring burstiness provides a highly efficient approach for analysing the composition of bitstreams and for excluding those that violate the IID assumption.

The remainder of this article is organised as follows: In Section 2 a selection of typical use cases for the application of random numbers across digital systems is presented. In Section 3 different generation methods of random sequences are given that form the basis for a number of random bit streams and their subsequent evaluation. In Section 4 the different random binary sequences are analysed with respect to their randomness and probabilistic characteristics. In Section 5 related results are presented and discussed. Finally, Section 6 provides concluding remarks.

## 2. Use Cases: Applications of Random Numbers Across Digital Systems

Random numbers generated by non-deterministic physical processes provide the entropy required by many digital systems. When the underlying process is quantum mechanical, the resulting output is referred to as quantum random numbers (QRNs). In practical systems, true random numbers (TRNs) are commonly used to seed cryptographically secure pseudorandom number generators (CSPRNGs), which expand a short random seed into a long pseudorandom sequence while preserving the security properties of the original seed.

High-quality entropy sources are required across a broad range of application domains. Cryptographic protocols rely on them for key generation, digital signatures, nonces, and initialisation vectors. In communication protocols such as TLS (Transport Layer Security), random nonces and session keys prevent replay, state-recovery, or brute-force attacks. For quantum cryptographic applications such as Quantum Key Distribution (QKD), TRNs are critical for real-time basis selection; if the choice of measurement basis is predictable, an eavesdropper can intercept photons without detection, compromising the quantum channel.

Mobile communication systems, including 5G and emerging 6G networks, use TRNs to support secure authentication and key establishment. Security studies emphasise that confidentiality, integrity, and availability requirements in 5G rely on cryptographic mechanisms whose resilience depends heavily on high-entropy random seeds and keys [12,13,14,15]. TRNs are particularly relevant in mitigating vulnerabilities during access and handover procedures. Furthermore, TRNG-based entropy sources play an important role in emerging 6G security frameworks, which must counter expanded attack surfaces arising from terahertz communication, dense IoT deployments (Internet of Things), artificial intelligence (AI)-managed networks, and quantum-capable adversaries.

In enterprise IT infrastructures, TRNs are employed for authentication, access control, and secure distributed services. They underpin the generation of cryptographically strong tags, tokens, session identifiers, and nonces in distributed systems. Their unpredictability is equally critical for producing unique identifiers in large-scale database systems to prevent collisions and inference attacks. TRNs also support secure key management, access-control mechanisms, multi-factor authentication tokens, and privacy-preserving techniques such as differential privacy.

Beyond security, stochastic simulations, including Monte Carlo methods and digital twins, require random inputs to avoid systematic bias. TRNs are used to produce stochastic inputs for system-generation processes to avoid deterministic artifacts that can distort outcomes. They support sensitivity analyses, risk assessments, stress-testing scenarios, and realistic modelling of noise and uncertainty in physical or socio-technical systems. Across these applications, the use of true randomness helps prevent statistical bias and increases the fidelity of simulated environments used in scientific research, engineering validation, and enterprise-scale decision-support systems.

Although these applications differ in their functional and performance requirements, they all rely on entropy sources that produce unpredictable random numbers with suitable statistical properties.

## 3. Generation of Random Bit Streams

### 3.1. Preamble

This section describes the generation methods for the different types of random sequences used in our analysis. The sequences are broadly categorised into two approaches: hardware-based (true random) and deterministic (pseudorandom) generation.

The hardware-based approach encompasses two distinct sources of true randomness: the ICTK VIA-PUF (Physically Unclonable Function), which exploits intrinsic manufacturing variations to derive device-unique cryptographic keys; and the IDQ Quantum RNG, which leverages quantum phenomena, specifically photon number fluctuations, to produce non-deterministic random bitstreams.

The deterministic approach includes the Linux kernel’s cryptographically secure pseudorandom number generator (CSPRNG), which is initially seeded and continuously reseeded with entropy from environmental sources such as device interrupts, timing variations, and input events, but remains a software-driven construct. Additionally, this approach employs linear and nonlinear feedback shift registers (LFSRs) to generate pseudorandom sequences. These sequences are reproducible given knowledge of the initial seed and the feedback polynomial.

A key contribution of this work is that not only are simulations and bitstreams generated by pseudorandom number generators analysed, but practically generated bit streams are also taken into account. The focus was placed on practically relevant approaches for generating bit streams that can be classified as random or at least nearly random according to results reported in the literature. For example, the bit streams generated by the IDQ Quantum RNG are based on physical quantum-optical randomness and are additionally certified according to the stringent NIST criteria. Such sequences provide valuable insights into the limits of randomness variation per block, demonstrating the practical applicability of the proposed gap-based analysis.

### 3.2. Hardware-Based Random Number Generation

#### 3.2.1. ICTK PUF

ICTK’s [16] VIA-PUF is a *p*hysically *u*nclonable *f*unction implemented using via structures that derives device unique cryptographic identities from the stochastic open/short-circuit behavior of via holes formed during semiconductor fabrication, leveraging uncontrollable process variations to create an immutable, chip-intrinsic fingerprint. Keys are generated internally through a KDF (key derivation function) applied to this unique via pattern, eliminating the need for external key injection and inherently preventing key leakage. Integrated as the core entropy source in ICTK’s hardware Root of Trust, VIA PUF supports secure key generation, certificate creation, and protected storage, and it also serves as a foundation for ICTK’s quantum resistant RoT (Root of Trust) SoC (System-on-Chip) architectures that combine VIA PUF with post quantum cryptography to ensure long term resilience against advanced and future threat models.

#### 3.2.2. IDQ Quantum RNG

The high-performance PCIe (Peripheral Component Interconnect Express) Quantum Random Number Generator is built on the latest Quantis QRNG technology (IDQ20MC1 chip) [17] of IDQuantique and provides true quantum entropy at rates of 40 Mbps and 240 Mbps. It incorporates an embedded NIST SP 800 90 A/B/C-compliant deterministic random bit generator [18,19,20] for standards aligned postprocessing, alongside on-chip health monitoring and failure detection mechanisms that ensure continuous operational integrity. Designed for seamless system integration, the device interfaces easily with Quantis software [17] and remains fully compatible with other Quantis PCIe and USB modules (Universal Serial Bus), enabling flexible deployment across diverse security critical environments.

### 3.3. Deterministic Pseudorandom Number Generation

Deterministic pseudorandom sequences are generated using software-based or algorithmic approaches, where the same initial state (seed) and parameters always produce the identical bitstream, making the sequence reproducible. This section covers both the Linux CSPRNG and feedback shift register-based sequences.

#### 3.3.1. Linux Cryptographically Secure Pseudorandom Number Generator

In our measurements, random numbers were obtained directly from the Linux kernel’s cryptographically secure pseudorandom number generator (CSPRNG) [21]. The Linux CSPRNG architecture accumulates entropy from unpredictable hardware driven events—including device interrupts, disk activity, and other environmental noise—which are continuously mixed into an internal entropy pool managed by the kernel. Once this pool is initialised, the kernel’s CSPRNG derives output bytes that are computationally infeasible to reverse engineer due to its cryptographic design. To retrieve random data, we used the standard user space interface /dev/urandom [21], which provides non blocking access to this CSPRNG output and is recommended for nearly all cryptographic applications because it remains secure even when new entropy input is temporarily limited. The resulting stream thus reflects kernel processed randomness originating from true environmental entropy sources, postprocessed through a conservative cryptographic generator.

#### 3.3.2. *m*-Sequence

Feedback shift registers (FSRs) generate deterministic pseudorandom sequences, where the same initial state (seed) and feedback function always produce an identical bitstream, making the sequence reproducible.

**Figure 1:**
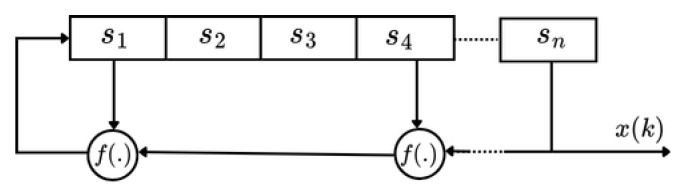
General structure of an *n*-stage feedback shift register (FSR).

Figure 1 illustrates the general structure of an *n*-stage feedback shift register. The register consists of *n* binary storage stages, denoted $s_{1},s_{2},\ldots ,s_{n}$, each holding a single bit. At each discrete time step (clock cycle), the contents of the register shift one position to the right: the bit from stage $s_{n}$ is output as x(k), while stages $s_{1}$ through $s_{n-1}$ move to $s_{2}$ through $s_{n}$, respectively. A new bit is then computed by the feedback function f(·), which takes the current stage contents as inputs, and is fed back into the first stage $s_{1}$. The feedback function *f* can be realised as a linear Boolean function (e.g., XOR of selected tap outputs) or as a nonlinear Boolean function (e.g., involving AND or OR operations), thereby enabling the generation of both linear and nonlinear pseudorandom sequences. The period and statistical properties of the resulting sequence depend on the length *n* of the shift register and the specific choice of the feedback function.

A maximum-length sequence (*m*-sequence) is a type of linear pseudorandom sequence generated by an LFSR with a primitive feedback polynomial [1,22]. Such sequences achieve the maximum possible period of $2^{n}-1$ for an *n*-stage register. This property makes them well-suited for applications that demand extended, uniformly distributed pseudorandom sequences [6].

In this work, an *m*-sequence was generated using an 11-stage LFSR with the primitive feedback polynomial $p\left(x\right)=x^{11}+x^{6}+x^{5}+x^{1}+1$. The register was initialised with the seed [0, 0, 0, 0, 0, 0, 0, 0, 0, 0, 1], and the output sequence was generated to a length of 1.6 million bits for analysis.

#### 3.3.3. Non-*m*-Sequence

A non-maximum-length sequence (non-*m*-sequence) is generated by an LFSR whose feedback polynomial is not primitive [1,23]. Consequently, the period of such a sequence is shorter than the maximum possible period of $2^{n}-1$ for an *n*-stage register. These sequences may exhibit different statistical properties compared to *m*-sequences [6].

In this work, a non-*m*-sequence was generated exemplarily by using a 10-stage LFSR with the feedback polynomial $p\left(x\right)=x^{10}+x^{9}+x+1$. The register was initialised with the seed [0, 0, 0, 0, 0, 0, 0, 0, 0, 1], and the output sequence was generated to a length of 1.6 million bits for analysis. It is important to note that this feedback polynomial decomposes as $p\left(x\right)=x^{10}+x^{9}+x+1=\left(x+1\right)\left(x^{9}+1\right)$ in GF(2). This decomposition indicates that the polynomial is not primitive. The resulting sequence has a period of 18 bits, significantly shorter than the maximum possible $2^{10}-1=1023$ bits.

To investigate the effect of the initial state, the same polynomial was tested with a different seed, [0, 0, 0, 0, 1, 0, 0, 0, 0, 1]. Furthermore, to verify that the observed non-IID characteristics are not an artifact of this particular polynomial, a different non-primitive polynomial, $p\left(x\right)=x^{11}+x^{10}+x^{7}+x^{5}+x+1$, was analysed with seed [0, 0, 0, 0, 0, 0, 0, 0, 0, 0, 1], resulting in a period of 126 bits. The results of these additional configurations are presented and discussed in Section 5.

#### 3.3.4. Nonlinear Sequence

A nonlinear feedback shift register (NFSR) employs a feedback function that includes nonlinear operations such as AND in addition to XOR. Unlike linear FSRs, nonlinear feedback can introduce correlations between consecutive bits, potentially leading to imbalanced sequences or bursty behavior.

In this work, a nonlinear sequence was generated exemplarily by using a 5-stage NFSR with the feedback function:

(1) $$f\left(x_{1},x_{2},x_{3},x_{4},x_{5}\right)=\left(x_{1}⊙x_{3}\right)⊕\left(x_{2}⊙x_{4}\right)⊕x_{5},$$

where ⊙ denotes the logical AND operation and ⊕ denotes the logical XOR operation in a finite field, e.g., Galois field GF(2) [24]. The register was initialised with the seed [1, 1, 0, 0, 1], and the output sequence was generated to a length of 1.6 million bits for analysis.

## 4. Evaluation of Random Bit Streams

This section presents the basis for a comprehensive evaluation of the random sequence sources described in Section 3, including both hardware-based random sources and deterministic pseudorandom sources. Each source was used to generate bitstreams of approximately 1.6 million bits, which were subsequently analysed using both the proposed gap-based methodology and selected tests from the NIST suite [7]. The evaluation focuses on detecting violations of the IID characteristics. As a mathematical basis, the gaps between neighbouring non-zero elements are analysed, e.g., [5,6].

The gaps between consecutive non-zero elements are characterised by the gap distribution function

(2) u(k)=P(Y≥k),k=0,1,2,3,…,

or, equivalently, by the gap density function

(3) v(k)=P(Y=k),k=0,1,2,3,….

The two functions are related via

(4) v(k)=u(k)−u(k+1).

For sequences where successive non-zero elements are independent, the ideal gap distribution function takes the form [25]

(5) $$u\left(k\right)={\left(1-p_{e}\right)}^{k},\;k=0,1,2,3,\ldots ,$$

where the parameter $p_{e}$ denotes the probability that a given element in the sequence is non-zero [5]. Substituting (5) into (4) yields the corresponding gap density function

(6) $$v\left(k\right)={\left(1-p_{e}\right)}^{k}\cdot p_{e}.$$

Equation (6) implies that, under the IID assumption, a non-zero element is immediately followed by another non-zero element (i.e., a gap of length k=0) with probability $v\left(0\right)=p_{e}$. In the particular case of a discrete uniform (DU) distribution of zero and non-zero elements, we have $p_{e}=0.5$ and consequently v(0)=0.5. Thus, fulfilment of the IID-DU condition necessarily requires v(0)=0.5.

The evaluation framework employs three complementary analytical approaches. First, the *monobit test* from the NIST suite [7] assesses the global distribution of 0s and 1s, providing a basic indication of statistical bias. Second, the proposed *gap density function* v(k) characterises the distribution of gaps between consecutive non-zero elements [5,6]. Third, the *burstiness parameter B* [9], defined as

(7) $$B=\frac{\sigma -m_{1}}{\sigma +m_{1}}\;,$$

where $m_{1}$ and σ represent the mean and standard deviation of the gap length, respectively. These statistical parameters are computed from the gap density function as follows:

(8) $$m_{1}=\sum_{k=0}^{\infty }k\;v\left(k\right)\;\mathrm{and}\;\sigma =\sqrt{\sum_{k=0}^{\infty }k^{2}\;v\left(k\right)-m_{1}^{2}}\;.$$

From (7) and (8) the burstiness level *B* is obtained. The burstiness parameter typically ranges from −1 to 1, where B=1 indicates a purely bursty sequence (i.e., all non-zero elements appear consecutively).

For an IID sequence with bit occurrence probability $p_{e}$, the ideal gap distribution follows the geometric distribution given in (5). For the specific case of a balanced IID-DU sequence with $p_{e}=0.5$, the ideal gap distribution has a mean of $m_{1}=1$ and a standard deviation of $\sigma =\sqrt{2}$. Substituting these values into (7) gives

(9) $$B=\frac{\sigma -m_{1}}{\sigma +m_{1}}=\frac{\sqrt{2}-1}{\sqrt{2}+1}=0.1716.$$

This theoretical value of *B* serves as the reference for burstiness throughout the subsequent analysis [26]. When sequences exhibit bursty behaviour, where non-zero elements tend to cluster together, significantly higher values of *B* are observed. This allows a clear differentiation between bursty and non-bursty sequences.

**Figure 2:**
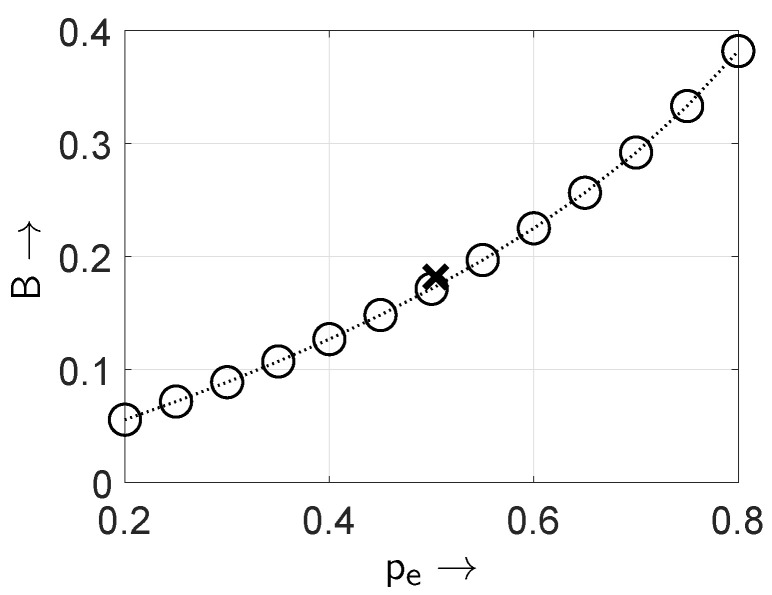
Theoretical *B* for different $p_{e}$ values. The cross marker indicates the *B* value (B=0.182) obtained from a reference sequence of N=10,000 bits produced using MATLAB’s randi([0 1]).

Figure 2 illustrates the theoretical relationship between the burstiness parameter *B* and the bit occurrence probability $p_{e}$. The cross marker indicates the simulation-based estimate (B=0.182) obtained from a 10,000-bit reference sequence using MATLAB R2021a randi([0 1]) function.

In order to illustrate the significance of the burstiness parameter *B*, we consider two exemplary binary sequences that share an identical conditional probability v(0)=0.5. This parameter indicates that the probability of observing a non-zero element immediately following another non-zero element is 50%, which satisfies one of the necessary conditions for an independent and identically distributed (IID) process. Despite this similarity, the sequences exhibit markedly different structural characteristics. The first sequence, for example, …110011000110011000…, exhibits gap lengths of zero with probability 50%, gap lengths of two with probability 25%, and gap lengths of three with probability 25%. For this sequence, the burstiness factor becomes B=0.0192. In contrast, the second sequence, …11101001110100…, exhibits gap lengths of zero with probability 50%, gap lengths of one with probability 25%, and gap lengths of two with probability 25%. The corresponding burstiness factor in this case is B=0.05.

This comparison demonstrates that, although both sequences share the same conditional probability v(0), their higher-order gap distributions differ significantly. Consequently, incorporating the full gap-length distribution, which is captured by the burstiness parameter *B*, enables a more nuanced classification of structural irregularities and anomalies.

## 5. Results and Discussion

A total of 1.6 million bits were generated for each source and subsequently divided into 20 non-overlapping blocks, each containing 80,000 bits. Each block was analysed using the aforementioned metrics. To ensure comprehensive characterisation, the analysis was performed in two ways: (1) per-block analysis of each individual 80,000-bit block, and (2) holistic analysis of the complete 1.6-million-bit sequence.

### 5.1. Linux Cryptographically Secure Pseudorandom Number Generator

Table 1 presents the average summary statistics for the Linux CSPRNG across 20 individual blocks of 80,000 bits each.

**Figure 3:**
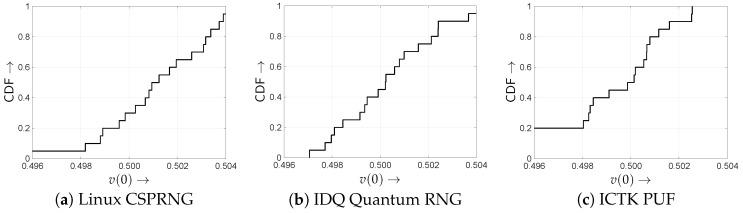
Cumulative distribution function (CDF) of v(0) across 20 blocks for each RNG source.

**Figure 4:**
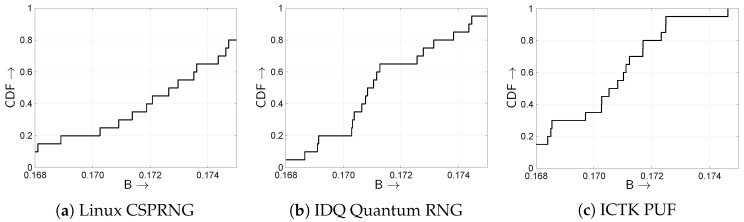
Cumulative distribution function (CDF) of burstiness parameter *B* across 20 blocks for each RNG source.

The Linux CSPRNG produces balanced sequences with mean values $p_{e}\approx 0.5$ and v(0)≈0.5, indicating that consecutive non-zero elements occur with the expected probability for an IID process. The burstiness parameter B≈0.172 is close to the theoretical IID reference value of 0.1716, confirming IID-DU behaviour. All blocks pass the monobit test with *p*-values above α=0.01. The cumulative distribution functions (CDFs) of v(0) and *B* (Figure 3a and Figure 4a) show narrow distributions around the ideal values, confirming consistent performance across blocks.

For the full 1.6 million-bit sequence, $p_{e}=0.5008$, v(0)=0.50117, and B=0.17246, all consistent with the per-block averages. The full-sequence monobit test yields *p*-value = 0.03465, which is lower than the per-block average of 0.4309 but remains above α=0.01. The per-block analysis averages 20 independent *p*-values obtained from individual 80,000-bit blocks. Several of these *p*-values are relatively high (e.g., close to 0.9), which pulls the average upward. In contrast, the full-sequence test applies a single test statistic to the entire 1.6 million-bit sequence, making it more sensitive to small imbalances accumulated across the entire sequence. The Linux CSPRNG exhibits a slightly larger block-to-block variation in *p*-values compared to the other sequences, which explains why this difference is more noticeable, although all blocks comfortably pass the test with *p*-value >α.

### 5.2. IDQ Quantum Random Number Generator

Table 2 presents the average summary statistics for the IDQ Quantum RNG across 20 individual blocks of 80,000 bits each.

The IDQ Quantum RNG exhibits near-ideal statistics with mean values $p_{e}\approx 0.5$, v(0)≈0.5, and B≈0.171, all consistent with the theoretical IID reference value of 0.1716. The CDFs in Figure 3b and Figure 4b confirm tight clustering around the ideal values. The full-sequence analysis yields $p_{e}=0.50029$, v(0)=0.50034, and B=0.17131, closely matching the per-block averages. The monobit test passes for both per-block (*p*-value = 0.5619) and full-sequence (*p*-value = 0.46316) analyses.

### 5.3. ICTK PUF

Table 3 presents the average summary statistics for the ICTK VIA-PUF across 20 individual blocks of 80,000 bits each.

The ICTK PUF produces statistically random sequences with mean values $p_{e}\approx 0.5$, v(0)≈0.5, and B≈0.170, close to the theoretical IID reference of 0.1716. The CDFs in Figure 3c and Figure 4c show distributions concentrated near the ideal values, though with slightly more spread than the other hardware source. The full-sequence analysis yields $p_{e}=0.49964$, v(0)=0.49915, and B=0.17033, confirming IID-DU behaviour. All blocks pass the monobit test.

### 5.4. *m*-Sequence

Table 4 presents the average summary statistics for the *m*-sequence across 20 individual blocks of 80,000 bits each.

The *m*-sequence exhibits mean values $p_{e}=0.5$, v(0)=0.5, and B=0.170, confirming IID-DU behaviour. The zero standard deviation is a consequence of its deterministic generation from a primitive polynomial. The full-sequence analysis yields $p_{e}=0.50024$, v(0)=0.5, and B=0.16954, closely matching the per-block averages. The monobit test passes for both per-block (*p*-value = 0.8913) and full-sequence (*p*-value = 0.050998) analyses.

### 5.5. Non-*m*-Sequence

Table 5 presents the average summary statistics for the non-*m*-sequence generated with $p\left(x\right)=x^{10}+x^{9}+x+1$ and seed [0, 0, 0, 0, 0, 0, 0, 0, 0, 1], calculated across 20 individual blocks of 80,000 bits each.

The non-*m*-sequence passes the monobit test with a mean *p*-value of 0.9867. However, the gap analysis reveals significant deviation from IID-DU behaviour: mean v(0)=0.889 indicates that 88.9% of non-zero elements are immediately followed by another non-zero element, far exceeding the ideal 50%. This clustering is captured by mean B=0.478, substantially higher than the IID reference of 0.1716. The full-sequence analysis yields $p_{e}=0.5$, v(0)=0.88889, and B=0.47759, confirming these findings.

To investigate the effect of the initial state, the same polynomial ($p\left(x\right)=x^{10}+x^{9}+x+1$) was tested with a different seed ([0, 0, 0, 0, 1, 0, 0, 0, 0, 1]), as summarised in Table 6. The sequence still passes the monobit test with a mean *p*-value of 0.9918. The gap analysis shows mean v(0)=0.667, indicating that 66.7% of non-zero elements are immediately followed by another non-zero element, and mean B=0.240, both above the IID reference values. The full-sequence analysis yields $p_{e}=0.5$, v(0)=0.66667, and B=0.24041, confirming the deviation from IID-DU behaviour.

To verify that the observed non-IID characteristics are not an artifact of the specific polynomial, a different non-primitive polynomial ($p\left(x\right)=x^{11}+x^{10}+x^{7}+x^{5}+x+1$) was analysed with seed [0, 0, 0, 0, 0, 0, 0, 0, 0, 0, 1], as summarised in Table 7. This sequence also passes the monobit test with a mean *p*-value of 0.9910. The gap analysis reveals mean v(0)=0.635 and mean B=0.299, both substantially higher than the IID reference values. The full-sequence analysis yields $p_{e}=0.5$, v(0)=0.63492, and B=0.29865, further confirming that non-IID behaviour is also observed for this different non-primitive polynomial.

These results demonstrate the benefit of the proposed burstiness parameter *B*, which successfully captures deviations from IID-DU behavior that remain undetected by the monobit test. Even when a sequence passes the monobit test due to a balanced distribution of 0s and 1s, the *B* parameter reveals underlying burstiness, highlighting the importance of analysing the inner structure of the sequence for comprehensive randomness evaluation.

### 5.6. Nonlinear Sequence

Table 8 presents the average summary statistics for the nonlinear sequence across 20 individual blocks of 80,000 bits each.

The nonlinear sequence exhibits clear non-IID behaviour with mean values $p_{e}=0.667$, v(0)=0.75, and B=0.268. The monobit test correctly detects this non-randomness with *p*-value = 0. Both v(0) and *B* indicate clustering of non-zero elements. The full-sequence analysis yields $p_{e}=0.66667$, v(0)=0.75, and B=0.26795, consistent with the per-block averages, with the monobit test producing *p*-value = 0.

These results demonstrate that the nonlinear sequence exhibits clear non-IID-DU behavior, characterised by imbalance ($p_{e}\neq 0.5$), high burstiness (B>0.176), and failure of the monobit test. This stands in contrast to the *m*-sequence and non-*m*-sequence, which are balanced and pass the monobit test.

**Figure 5:**
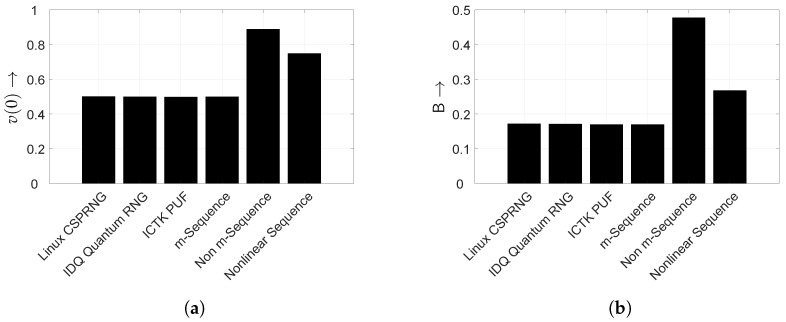
Comparison of (**a**) the probability of consecutive non-zero elements v(0) and (**b**) the burstiness parameter *B* for all six evaluated sequences.

Table 9 and Figure 5 summarise the results across all sequences. The hardware-based sources (IDQ Quantum RNG and ICTK PUF) and the deterministic sequences (Linux CSPRNG and *m*-sequence) exhibit v(0)≈0.5 and B≈0.171, consistent with IID-DU behaviour. In contrast, the non-*m*-sequence and the nonlinear sequence show elevated v(0) and *B* values, indicating non-IID behaviour. In particular, the non-*m*-sequence demonstrates that the proposed gap-based approach can detect non-IID structure even when the monobit test passes.

## 6. Conclusions

Across cryptography, mobile networks, enterprise IT systems and simulation environments, true random numbers provide the foundation for unpredictability, robustness and security. Their ability to produce high-entropy, non-deterministic values protects systems from inference, correlation, and state-recovery attacks while ensuring the integrity of computational and analytical processes. As digital infrastructure grows increasingly complex and interconnected, the reliance on strong entropy sources intensifies, positioning TRNGs as indispensable components of modern secure and resilient system design.

In this work alternative test mechanisms for detecting non-IID behaviour have been evaluated. The proposed testing procedures are based on the gaps between neighbouring non-zero elements and the calculated gap density function. Together with the burstiness parameter, this allows a low-complexity detection whether or not a given randomised bit sequence exhibits the IID-DU characteristic.

The results show that true random number sequences and the *m*-sequence adhere to the IID-DU assumption, as evidenced by v(0)≈0.5 and B≈0.17. The non-*m*-sequence and the nonlinear sequence, however, deviate significantly with v(0)>0.5 and B>0.17. These findings highlight the importance of analysing the gap density function and the burstiness parameter in detecting non-IID behaviour that remains hidden from the monobit test.

## Figures and Tables

**Table 1:** Average summary statistics for Linux CSPRNG across 20 blocks (80 kbits each).

Metric	Mean	Standard Deviation
$p_{e}$	0.5008	0.0018
*p*-value	0.4309	0.3193
v(0)	0.5012	0.0025
*B*	0.1724	0.0031

**Table 2:** Average summary statistics for IDQ Quantum RNG across 20 blocks (80 kbits each).

Metric	Mean	Standard Deviation
$p_{e}$	0.5003	0.0017
*p*-value	0.5619	0.3059
v(0)	0.5003	0.0020
*B*	0.1713	0.0023

**Table 3:** Average summary statistics for ICTK VIA-PUF across 20 blocks (80 kbits each).

Metric	Mean	Standard Deviation
$p_{e}$	0.49964	0.0016
*p*-value	0.5346	0.28977
v(0)	0.4992	0.00252
*B*	0.1703	0.00213

**Table 4:** Average summary statistics for *m*-sequence across 20 blocks (80 kbits each).

Metric	Mean	Standard Deviation
$p_{e}$	0.5000	0.0000
*p*-value	0.8913	0.0339
v(0)	0.5000	0.0000
*B*	0.1700	0.0000

**Table 5:** Average summary statistics for non-*m*-sequence ($p\left(x\right)=x^{10}+x^{9}+x+1$, seed [0, 0, 0, 0, 0, 0, 0, 0, 0, 1]) across 20 blocks (80 kbits each).

Metric	Mean	Standard Deviation
$p_{e}$	0.5000	0.0000
*p*-value	0.9867	0.0076
v(0)	0.8890	0.0000
*B*	0.4780	0.0000

**Table 6:** Average summary statistics for non-*m*-sequence ($p\left(x\right)=x^{10}+x^{9}+x+1$, seed [0, 0, 0, 0, 1, 0, 0, 0, 0, 1]) across 20 blocks (80 kbits each).

Metric	Mean	Standard Deviation
$p_{e}$	0.5000	0.0000
*p*-value	0.9918	0.0067
v(0)	0.6667	0.0000
*B*	0.2404	0.0000

**Table 7:** Average summary statistics for non-*m*-sequence ($p\left(x\right)=x^{11}+x^{10}+x^{7}+x^{5}+x+1$, seed [0, 0, 0, 0, 0, 0, 0, 0, 0, 0, 1]) across 20 blocks (80 kbits each).

Metric	Mean	Standard Deviation
$p_{e}$	0.5000	0.0000
*p*-value	0.9910	0.0074
v(0)	0.6349	0.0000
*B*	0.2987	0.0000

**Table 8:** Average summary statistics for nonlinear sequence across 20 blocks (80 kbits each).

Metric	Mean	Standard Deviation
$p_{e}$	0.6670	0.0000
*p*-value	0.0000	0.0000
v(0)	0.7500	0.0000
*B*	0.2680	0.0000

**Table 9:** Comparative summary of average v(0), *B*, and *p*-values for all evaluated sequences.

Sequence	v(0)	*B*	*p*-Value
IDQ Quantum RNG	0.5003	0.1713	0.5619
ICTK PUF	0.4992	0.1703	0.5346
Linux CSPRNG	0.5012	0.1724	0.4309
*m*-Sequence	0.5000	0.1700	0.8913
Non-*m*-Sequence	0.8890	0.4780	0.9867
Nonlinear Sequence	0.7500	0.2680	0.0000

## Data Availability

The datasets used in this study were either generated using the algorithms described therein or obtained via standard commercially available hardware. Consequently, no external datasets are provided.

## Abbreviations

The following abbreviations are used in this manuscript:

5G	Fifth Generation of Mobile Networks
6G	Sixth Generation of Mobile Networks
AI	Artificial Intelligence
CSPRNG	Cryptographically Secure Pseudorandom Number Generator
IID	Independent and Identically Distributed
IoT	Internet of Things
IT	Information Technology
KDF	Key Derivation Function
LFSR	Linear Feedback Shift Register
NIST	National Institute of Standards and Technology
PCIe	Peripheral Component Interconnect Express
PUF	Physically Unclonable Function
QKD	Quantum Key Distribution
QRN	Quantum Random Number
RoT	Root of Trust
SoC	System-on-Chip
TLS	Transport Layer Security
TRN	True Random Number
TRNG	True Random Number Generator
USB	Universal Serial Bus
